# Comparison of the effects of remimazolam tosylate and propofol on immune function and hemodynamics in patients undergoing laparoscopic partial hepatectomy: a randomized controlled trial

**DOI:** 10.1186/s12871-024-02589-4

**Published:** 2024-06-10

**Authors:** Qi Xing, Xuelong Zhou, Yin Zhou, Chonglong Shi, Wenjie Jin

**Affiliations:** Department of Anesthesiology and Perioperative Medicine, the First Affiliated Hospital of Nanjing Medical University, 300 Guangzhou Road, Nanjing, 210029 China

**Keywords:** Remimazolam tosylate, Propofol, Partial hepatectomy, Immune function, Hemodynamics

## Abstract

**Background:**

Laparoscopic partial hepatectomy inevitably decrease patient immune function. Propofol has been shown to have immunomodulatory effects but is associated with hemodynamic side effects. Despite studies showing a negligible impact of remimazolam tosylate on hemodynamics, it has not been reported for partial hepatectomy patients. Its influence on immune function also remains unexplored. This study sought to investigate the differences in immune function and intraoperative hemodynamics between patients who underwent laparoscopic partial hepatectomy with remimazolam tosylate and those who underwent laparoscopic partial hepatectomy with propofol.

**Methods:**

This was a single-center, randomized controlled trial involving 70 patients, who underwent elective laparoscopic partial hepatectomy. The patients were randomly divided into two groups: the remimazolam group (group R) and the propofol group (group P). In this study, the primary outcomes assessed included the patient’s immune function and hemodynamic parameters, and the secondary outcomes encompassed the patient’s liver function and adverse events.

**Results:**

Data from 64 patients (group R, *n* = 31; group P, *n* = 33) were analyzed. The differences in the percentages of CD3^+^, CD4^+^, CD8^+^, and NK cells and the CD4^+^/CD8^+^ ratio between the two groups were not statistically significant at 1 day or 3 days after surgery. Compared with those in group P, the MAP and HR at T2 and the MAP at T1 in group R were significantly increased(*P* < 0.05). The differences in HR and MAP at T0, T3, T4, T5, T6, and T7 and HR at T1 between the two groups were not statistically significant. There were no differences in liver function or adverse effects between the two groups, suggesting that remimazolam tosylate is a safe sedative drug(*P* > 0.05).

**Conclusion:**

The effects of remimazolam tosylate on the immune function of patients after partial hepatectomy are comparable to those of propofol. Additionally, its minimal effect on hemodynamics significantly decreases the incidence of hypotension during anesthesia induction, thereby enhancing overall perioperative safety.

**Trial registration:**

The trial was registered on May 9, 2022 in the Chinese Clinical Trial Registry, registration number ChiCTR2200059715 (09/05/2022).

## Introduction

Liver tumors include both malignant (cancerous) and benign (non-cancerous) types, with hepatocellular carcinoma being the most common in the former, accounting for approximately 80% of cases [1], and hemangiomas and focal nodular hyperplasia being the most common in the latter [2]. Partial hepatectomy is the treatment of choice for liver tumors [3]. Laparoscopic hepatectomy has become an increasingly popular option for surgeons over the past decade due to advances in hepatectomy safety and laparoscopic equipment [4]. The liver plays a crucial role in the differentiation of immune cells. Partial hepatectomy, while essential for tumor treatment, results in a reduction in the body’s immune function. This decrease may lead to delayed incision healing and an increased risk of postoperative infections, sepsis, and other complications [5, 6]. Changes in immune function after partial hepatectomy seriously affect patient prognosis. Therefore, selecting anesthetic drugs that have minimal impact on immune function while ensuring safety is imperative. As a traditional intravenous anesthetic, propofol is characterized by its fast onset and short duration. However, it is associated with adverse reactions, including post-waking dizziness, respiratory and circulatory depression, injection pain, accumulation with prolonged infusion, and metabolic acidosis [7, 8]. Patients with liver tumors often have varying degrees of cirrhosis, and structural changes in the liver significantly reduce blood flow, which in turn affects propofol metabolism, thereby increasing the risk of hypotension [9]. In comparison, remimazolam tosylate offers advantages such as minimal circulatory system inhibition, mild injection pain, absence of drug accumulation in the body, effective reversal by the benzodiazepine antagonist flumazenil, and the ability to shorten the patient’s recovery room stay [10]. However, the effects of remimazolam tosylate for partial hepatectomy on immune function and circulatory stability are unclear. Therefore, this study aimed to compare the effects of remimazolam tosylate and propofol on postoperative immune function as well as intraoperative hemodynamics in patients who underwent partial hepatectomy, providing valuable insights for clinical application.

## Materials and methods

This prospective randomized controlled trial was approved by the Ethics Committee of the First Affiliated Hospital of Nanjing Medical University on March 15, 2022 (approval number: 2022-SR-035) and registered at the Chinese Clinical Trial Registry (registration number: ChiCTR2200059715). All patients signed written informed consent. A total of 70 patients with liver-occupying lesions scheduled for laparoscopic partial hepatectomy from May 2022 to May 2023 were enrolled.

### Inclusion criteria

Age 18–70 years; BMI 18.5–27.9 kg/m^2^; American Society of Anesthesiologists (ASA) I or II; preoperative transaminase levels less than 2.5 times normal; cirrhotic patients with Child-Pugh classification A.

### Exclusion criteria

Histories of chemotherapy, radiotherapy, interventional therapy, immune-assisted therapy; thrombosis, bile duct obstruction, portal vein or hepatic artery thrombosis; cardiac, pulmonary, renal and other vital organ insufficiency; autoimmune disease or adrenocortical insufficiency; prolonged use of sedative and analgesic drugs; allergy to the drugs used in this study; psychiatric disorders; long-term alcoholism or a history of heavy alcohol consumption in the recent past.

### Termination criteria

Intermediate open surgery; intraoperative hemorrhage or air embolism; postoperative complications such as fever and infection; serious electrolyte disorders in blood gas analysis (e.g., serum K^+^≤2.5 mmol/L or serum K^+^≥5.3 mmol/L; serum Na^+^≤130 mmol/L or serum Na^+^≥150 mmol/L) or other serious acid-base balance disorders (pH < 7.25 or pH > 7.55).

### Study design

In this randomized controlled study, eligible patients were randomly divided into two groups according to the allocation sequence generated by the computer program: the remimazolam group (group R) and the propofol group (group P), with 35 patients in each group. Remimazolam tosylate (0.2 mg/kg) was injected intravenously in group R and propofol (2 mg/kg) was injected intravenously in group P to induce anesthesia. During the maintenance of anesthesia, patients in group R were infused with remimazolam tosylate (0.4–1.2 mg/kg/h), while patients in group P were infused with propofol (4–10 mg/kg/h). The patient, the surgeon, and the researcher who recorded the data and conducted the postoperative follow-up were unaware of the anesthesia regimen.

### Laparoscopic partial hepatectomy

The surgeries were completed by the same group of tertiary physicians from the Hepatobiliary Center of the First Affiliated Hospital of Nanjing Medical University. Regular hepatic segmental resection was performed laparoscopically according to the anatomical characteristics of intrahepatic blood vessels and bile ducts. Symptomatic supportive treatment and care, including hepatoprotection, anti-infection, acid-suppression and gastric protection, and nutritional enhancement, were uniformly provided.

### Anesthesia procedures

Patients in both groups routinely fasted for 8 h before surgery. After the patients were admitted to the operating room, they were given oxygen via a mask (with a partial pressure of 100% oxygen, and an oxygen flow rate of 6 L/min), intravenous access was established, and electrocardiogram (ECG), heart rate (HR), pulse oxygen saturation (SPO_2_) and the bispectral index (BIS) were routinely monitored. The left radial artery was punctured, the right internal jugular vein was punctured and cannulated, and the mean arterial pressure (MAP) and central venous pressure (CVP) were monitored. Anesthesia induction involved intravenous injection of remimazolam tosylate (0.2 mg/kg) for Group R and propofol (2 mg/kg) for Group P. When the BIS value decreased to 60, both groups received intravenous sufentanil (0.3 µg/kg) and cisatracurium (0.2 mg/kg). Additional doses of remimazolam tosylate(0.05 mg/kg) for Group R and propofol (0.5 mg/kg) for Group P could be administered, which were repeated up to three times until the BIS value was less than 60. Subsequently, tracheal intubation was performed under visual laryngoscopy, followed by connection to the anesthesia machine and a switch to volume-controlled ventilation mode with air-oxygen mixing (1 L/min each). The parameters were set as follows: tidal volume (VT) 6–8 ml/kg, respiratory rate (RR) 10–16 times/min, inspiration-to-expiration ratio (I: E) 1:2, and end-tidal carbon dioxide (P_ET_CO_2_) maintained at 35–45 mmHg. Anesthesia maintenance: Group R was intravenously injected with remimazolam tosylate (0.4–1.2 mg/kg/h), and group P was intravenously injected with propofol (4–10 mg/kg/h). Patients in both groups were administered remifentanil (0.1–0.2 µg/kg/min) and cisatracurium (0.1–0.2 mg/kg/h). Additional sufentanil was added to 0.6 to 0.8 µg/kg before the start of surgery. Intraoperatively, the BIS value of 40–60 was maintained by adjusting the pumping rate of the remimazolam tosylate and propofol, and the CVP was maintained at 0–5 cmH_2_O before liver resection. Oxycodone (5 mg) was added to the specimen at the time of specimen retrieval in both groups, and 1–5 mg of oxycodone was added intraoperatively at the discretion of the patients. If the intraoperative MAP was less than 65 mmHg or MAP was less than 70% of the basal value, phenylephrine (0.1 mg) or ephedrine (6 mg) was intermittently injected, depending on whether the HR was less than 60 beats/min. If the number of vasoactive drugs used was greater than 3 times in 30 min, norepinephrine was administered via the central vein. Approximately 30 min before the end of the surgery, the pumping of muscarinic drugs was stopped, and the rest of the drugs were stopped immediately after the surgery. The patients were transferred to the Post-Anesthesia Care Unit (PACU), and when the patient’s spontaneous respiration was restored, group R received intravenous flumazenil (0.5 mg). When the patient met the indications for extubation, this procedure was performed by a dedicated nurse anesthetist in the PACU.

### Data collection

The primary endpoints consisted of cellular immune function and hemodynamic indices. Peripheral venous blood samples (2 ml) were collected from patients before surgery, 1d after surgery, and 3d after surgery, respectively. The levels of CD3^+^, CD4^+^, CD8^+^, CD4^+^/CD8^+^, and NK cells were detected by flow cytometry at the laboratory of the Department of Oncology of the First Affiliated Hospital of Nanjing Medical University. MAP and HR were recorded at each time point as follows: before induction of anesthesia (T0), at a BIS value of 60 (T1), at the time of tracheal intubation (T2), at the time of skin incision (T3), at the beginning of hepatic portal block (T4), 10 min after the first hepatic portal block (T5), at the end of hepatic portal block (T6), and at the time of extubation (T7).

The secondary outcomes were liver function markers and incidence of adverse effects. Peripheral venous blood samples (2 ml) were collected from the patients before the operation, 1d after the operation, and 3d after the operation to test alanine aminotransferase (ALT), aspartate aminotransferase (AST) and lactic dehydrogenase (LDH) levels via the rate method in the biochemistry laboratory of the First Affiliated Hospital of Nanjing Medical University. Additionally, the incidence of postoperative nausea, vomiting, hypoxemia, and hypotension was recorded.

### Statistical analysis

The sample size was calculated by PASS15.0 software. The predefined main index of this study was the CD4^+^/CD8^+^ ratio at 1 d postoperatively, which was 1.76 ± 0.43 in the group R and 1.41 ± 0.19 in the group P according to the results of the pre-experiment (10 patients in each group). With α = 0.05 and 1-β = 0.9, a required sample size of 28 cases for each group was determined, accounting for an expected 20% loss-to-follow-up rate. Therefore, the final sample included 35 patients in each group.

IBM SPSS Statistics 26.0 software was used for data analysis. Continuous measurement data was expressed as the mean ± standard deviation, and statistical analyses were performed by independent sample t-test and repeated measures ANOVA; count data were expressed as numbers (percentages), and statistical analyses were performed by the chi-square test and Fisher’s exact probability method. *P* < 0.05 was considered to indicate a statistically significant difference.

## Results

**Fig. 1:**
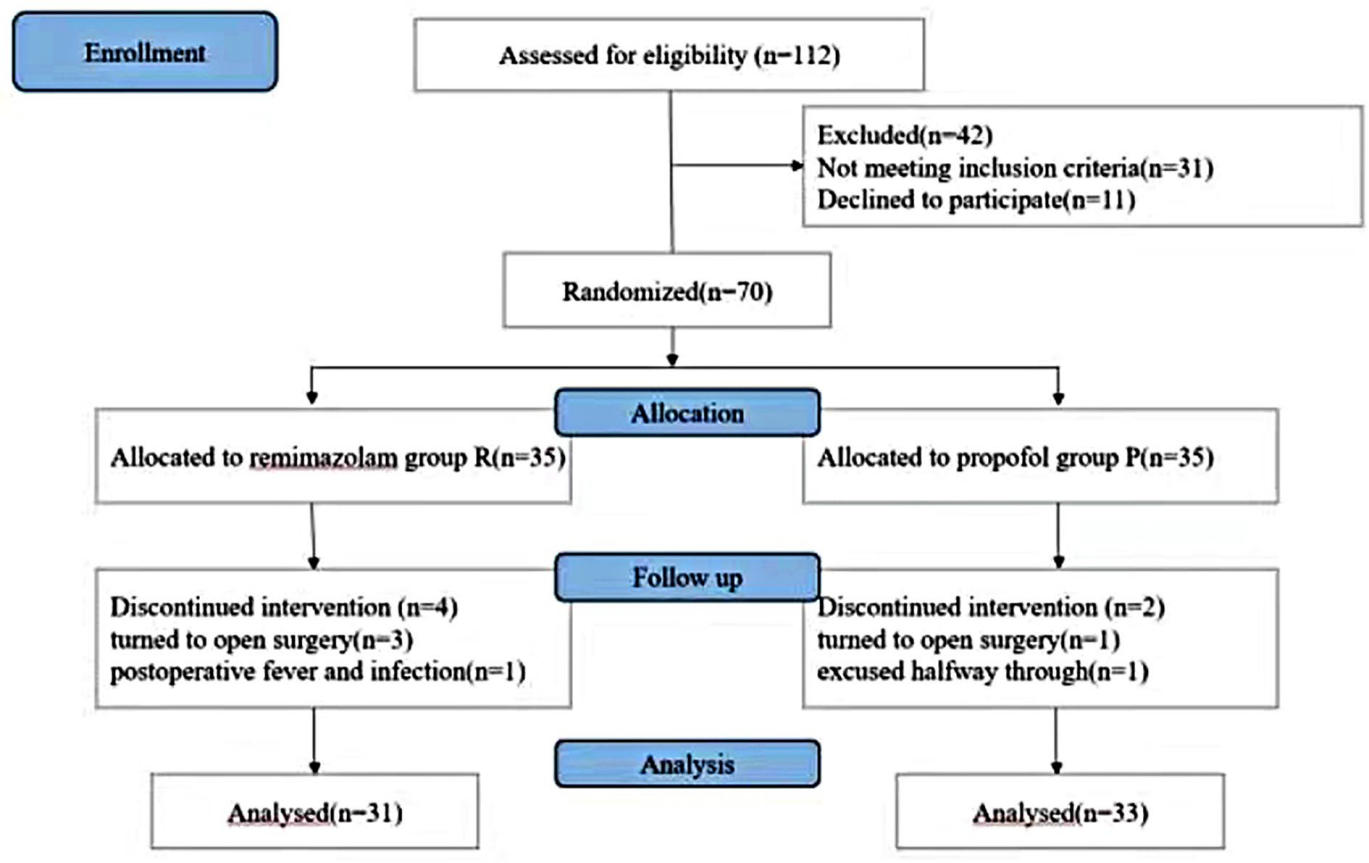
Flowchart of the patients

A total of 70 patients were initially included in this study. Three patients in group R were excluded for intraoperative conversion to open surgery, and one patient was excluded for postoperative fever and infection. One patient in group P was excluded for intraoperative conversion to open surgery, and one patient requested to be excused halfway through. Overall, 64 patients were recruited, with 31 in group R and 33 in group P. The flowchart of the patients is shown in Figure (Fig. 1).

There was no statistical difference between the two groups in terms of sex, age, BMI, ASA classification, intraoperative input, blood loss, hepatic portal block time, operation time or pathological type (*P* > 0.05)(Table 1).

**Table 1:** Basic characteristics and surgical information of the patients

	Group R(*n* = 31)	Group P(*n* = 33)	*P*-value
Sex (male/female)	17/14	19/14	0.825
Age (years)	54.8 ± 11.0	53.9 ± 11.7	0.746
BMI (kg/m^2^)	23.5 ± 2.8	24.6 ± 2.8	0.108
ASA status (ǀ/ǁ)	6/25	7/26	0.854
intraoperative input(ml)	1471.0 ± 550.0	1653.9 ± 454.3	0.151
blood loss(ml)	267.7 ± 173.9	315.2 ± 185.6	0.297
hepatic portal block time(min)	41.1 ± 26.7	53.7 ± 26.7	0.064
operation time(min)	148.6 ± 63.4	177.0 ± 52.6	0.055
Malignancy	11(35.5%)	18(54.5%)	0.126

### Immune function

The intra-group comparisons of CD3^+^, CD4^+^, CD8^+^, and NK cell levels and CD4^+^/CD8^+^ ratios in group R and group P showed substantial differences in cell levels at each time point (*P* < 0.05), although there was no statistical difference between groups (*P* > 0.05). Compared with the preoperative period, CD3^+^ and CD4^+^ cell levels, as well as the CD4^+^/CD8^+^ ratio, were significantly lower, and NK cell levels were significantly higher in both groups at 1d postoperatively; CD8^+^ cell levels were significantly lower, while the CD4^+^/CD8^+^ ratio was significantly higher in group R at 3d postoperatively, and NK cell levels were significantly lower in both groups at 3d postoperatively, with statistically significant differences (*P* < 0.05). Compared with 1d postoperatively, CD3^+^ and CD4^+^ cell levels and the CD4^+^/CD8^+^ ratio were significantly higher, and NK cell levels were significantly lower in both groups at 3d postoperatively (*P* < 0.05) (Table 2).

**Table 2:** Indicators of cellular immunity at different time points

Indicators		Preoperative	1d postoperative	3d postoperative
CD3^+^(%)	Group R (*n* = 31)	69.8 ± 9.6	60.4 ± 9.7^a^	69.1 ± 9.8^b^
	Group P (*n* = 33)	70.5 ± 8.0	61.5 ± 10.3^a^	70.6 ± 8.2^b^
	*p*-value	0.744	0.647	0.528
CD4^+^(%)	Group R (*n* = 31)	41.7 ± 9.0	32.5 ± 6.7^a^	43.0 ± 8.5^b^
	Group P (*n* = 33)	42.1 ± 7.2	32.3 ± 8.7^a^	42.6 ± 8.1^b^
	*p*-value	0.834	0.916	0.824
CD8^+^(%)	Group R (*n* = 31)	23.9 ± 7.4	22.8 ± 7.1	21.8 ± 6.8^a^
	Group P (*n* = 33)	24.2 ± 5.4	24.9 ± 7.6	23.9 ± 5.9
	*p*-value	0.829	0.255	0.196
CD4^+^/CD8^+^	Group R (*n* = 31)	2.0 ± 0.9	1.6 ± 0.8^a^	2.2 ± 1.1^ab^
	Group P (*n* = 33)	1.8 ± 0.6	1.5 ± 0.6^a^	1.9 ± 0.7^b^
	*p*-value	0.395	0.341	0.187
NK cell(%)	Group R (*n* = 31)	14.2 ± 7.8	19.2 ± 9.1^a^	10.6 ± 5.9^ab^
	Group P (*n* = 33)	15.7 ± 8.4	20.3 ± 9.5^a^	12.2 ± 6.8^ab^
	*p*-value	0.469	0.643	0.320

^a^*P* < 0.05 compared with preoperative in each group; ^b^*P* < 0.05 compared with 1d postoperative in each group

### Hemodynamics

**Fig. 2:**
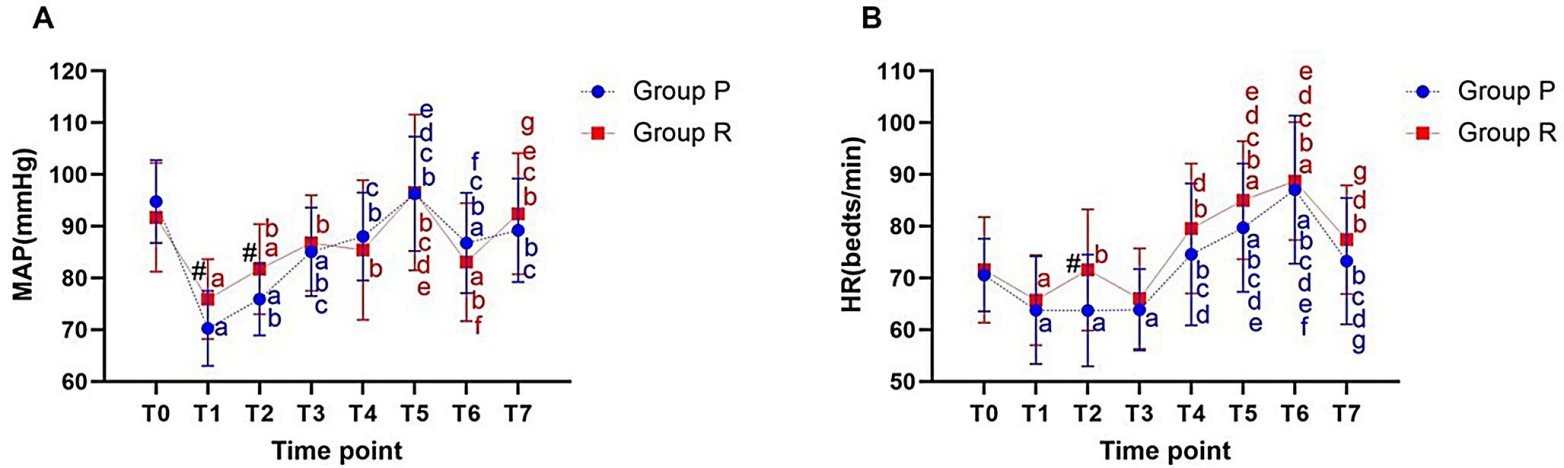
(**A**) Changes in MAP during surgery; (**B**) Changes in HR during surgery. (T0) before induction of anesthesia; (T1) at a BIS value of 60; (T2) at the time of tracheal intubation; (T3) at the time of skin incision; (T4) at the beginning of hepatic portal block; (T5)10 min after the first hepatic portal block; (T6) at the end of hepatic portal block; (T7) at the time of extubation. #*p* < 0.05 compared between two groups; ^a^*P* < 0.05 compared with T0 in each group; ^b^*P* < 0.05 compared with T1 in each group; ^c^*P* < 0.05 compared with T2 in each group; ^d^*P* < 0.05 compared with T3 in each group; ^e^*P* < 0.05 compared with T4 in each group; ^f^*P* < 0.05 compared with T5 in each group; ^g^*P* < 0.05 compared with T6 in each group

Compared with group P, the MAP at T1 and T2 and the HR at T2 were significantly higher in group R, and the difference was statistically significant (*P* < 0.05), while the differences in HR, MAP at T0, T3, T4, T5, T6 and T7, and HR at T1 were not statistically significant (*P* > 0.05). Compared with T0, the MAP at T1, T2, and T6 and the HR at T1 were significantly lower, while the HR at T5 and T6 were significantly higher in group R; the MAP at T1, T2, T3, andT6 and the HR at T1, T2, andT3 were significantly lower, while the HR at T5 and T6 were significantly higher in group P, and the difference was statistically significant (*P* < 0.05) (Fig. 2).

### Liver function

Compared with group P, there were no statistically significant differences in the ALT, AST, or LDH levels in group R during the preoperative period, 1d postoperatively, or 3d postoperatively(*P* > 0.05) (Table 3).

**Table 3:** Liver function indicators at different time points

Indicators		Preoperative	1d postoperative	3d postoperative
ALT(U/L)	Group R (*n* = 31)	26.4 ± 18.6	304.2 ± 344.1^a^	183.1 ± 182.7^ab^
	Group P (*n* = 33)	22.8 ± 12.7	302.9 ± 202.3^a^	192.6 ± 140.0^ab^
	*p*-value	0.357	0.985	0.815
AST(U/L)	Group R (*n* = 31)	26.0 ± 9.8	289.0 ± 323.1^a^	76.1 ± 66.3^ab^
	Group P (*n* = 33)	22.2 ± 5.5	290.9 ± 200.5^a^	78.5 ± 64.4^ab^
	*p*-value	0.061	0.977	0.880
LDH(U/L)	Group R (*n* = 31)	186.1 ± 42.0	418.7 ± 293.3^a^	217.4 ± 41.4^ab^
	Group P (*n* = 33)	178.6 ± 33.5	393.9 ± 163.1^a^	231.0 ± 61.1^ab^
	*p*-value	0.432	0.676	0.305

^a^*P* < 0.05 compared with preoperative in each group; ^b^*P* < 0.05 compared with 1d postoperative in each group

### Side effects

Compared with group P, the incidence of adverse reactions such as nausea, vomiting, hypoxemia, and hypotension tended to decrease among patients in group R postoperatively, but there was no statistical difference (*P* > 0.05) (Table 4).

**Table 4:** Incidence of postoperative adverse reactions

	Group *R*	Group *P*	*p*-value
nausea	7 (22.6%)	9 (27.3%)	0.665
vomiting	5 (16.1%)	6 (18.2%)	0.828
hypoxemia	4 (12.9%)	6 (18.2%)	0.734
hypotension	1 (3.2%)	3 (9.1%)	0.614

## Discussion

In this study, the impact of laparoscopic partial hepatectomy under propofol and remimazolam tosylate anesthesia on patients’ immune function and hemodynamics was explored. Both demonstrated comparable effects on cellular immune function, while remimazolam tosylate exhibited enhanced hemodynamic stability.

Currently, laparoscopic partial hepatectomy is the first-line treatment for liver tumors, but surgical intervention and anesthetic process inevitably cause a decrease in immune function. Therefore, reasonable selection of anesthetic drugs in the perioperative period to minimize adverse effects on immune function is conducive to improving patient prognosis. Immunosuppression is recognized as an important factor affecting recovery and survival after partial hepatectomy. The immune changes occurring in the perioperative period include the activation of the hypothalamic-pituitary-adrenal (HPA) axis by surgical trauma and the generation of a series of neuroendocrine responses, which induce the release of cortisol and suppress the host immune response; on the other hand, after the activation of the sympathetic nervous system (SNS), the release of catecholamines and prostaglandin E2 (PGE2) acts as an immune-suppressing factor [11]. The magnitude of surgical trauma, i.e., the intensity of the stress response, determines to some extent the degree of immunosuppression [11].

As central players in the immune system, T-lymphocyte subsets play important role in cellular immunity. T cells are classified into helper T-lymphocytes (THs) and cytotoxic T-lymphocytes (CTLs) based on their function and main surface markers (CD4^+^ and CD8^+^). CD3 is a surface molecule present on all T cells and responds to the overall level of cellular immunity [12]. CD4^+^ T cells can proliferate to activate other types of immune cells, generating a direct immune response or providing a secondary helper for other lymphocytes; CD8^+^ T cells, also known as killer T cells, carry out the immune response mainly by recognizing and killing target cells of infectious pathogens and are one of the key components of the cell-mediated immune response [13, 14]. The CD4^+^/CD8^+^ ratio can be used as an important indicator to assess the state of the body’s immune function and is important for guiding clinical treatment and prognosis judgment. A decrease in the CD4^+^/CD8^+^ ratio may indicate that the immune system’s ability to respond to disease or infection is weakened, which increases the risk of disease progression and poor prognosis [15]. NK cells are part of the innate immune system and destroy pathogen-infected and tumor cells by releasing cytotoxic particles that promote protein hydrolytic cleavage of harmful cells, leading to apoptosis [16]. NK cells are innate cytotoxic lymphocytes with adaptive immune characteristics and are considered to bridge the gap between innate and adaptive immunity. There is close cooperation between NK cells and CD8 T cells, which play important roles in immune function and disease pathogenesis.

Propofol is a short-acting intravenous anesthetic that produces a sedative-hypnotic effect by acting on central gamma-aminobutyric acid (GABA) receptors and is characterized by a controllable degree of anesthesia, rapid induction, quick recovery, and few adverse effects. Recent studies have shown that propofol also regulates immune function. Propofol anesthesia was chosen for surgeries such as radical hysterectomy, radical nephrectomy, partial hepatectomy, and radical gastrectomy for gastric cancer, with fewer effects on cellular immune function than sevoflurane, which may be related to the reduction in serum catecholamine and cortisol concentrations [17–20]. Liu et al. demonstrated that propofol is superior to isoflurane and enflurane in inhibiting serum IL-8 secretion and increasing IL-10 secretion and can be considered a better anesthetic for suppressing the inflammatory response to trauma [21]. Unlike volatile anesthetics, propofol stimulates NK cell activity, reduces pro-inflammatory cytokine levels, and inhibits COX-2 and PGE2 function [11, 22]. In addition, propofol does not affect the ratio of Th1 to Th2 cells, thereby attenuating surgery-induced immunosuppression [23]. Thus, propofol, the most commonly used intravenous anesthetic in clinical practice, has significant advantages over inhaled anesthetics in terms of immunomodulation. The effects of remimazolam tosylate, the latest approved intravenous anesthetic for the induction and maintenance of general anesthesia, on immune function have not yet been studied. Remimazolam tosylate is a new ultrashort-acting benzodiazepine (BDZ). The action of BDZ depends on the activation of binding sites, such as the central benzodiazepine binding site (CBBS) and the peripheral translocator protein (TSPO). The CBBS is found in several regions of the central nervous system and is a component of the GABA_A_ receptor. Binding to this site enhances GABAergic neurotransmission and affects the neuroendocrine response by counteracting stress-induced overactivation of the HPA axis and inhibiting the secretion of corticotropin-releasing hormone (CRH) [24, 25]. TSPO is present in lymphocytes, macrophages, monocytes, and other immune cells, and BDZ bound to TSPO can affect cellular immune function by inhibiting cytokine secretion [26–28], modifying cell proliferation, and affecting immune cell migration [29] and cellular phagocytic activity [30]. Therefore, similar to propofol, remimazolam tosylate may also have an immunomodulatory function. The results of this study showed that in the postoperative period from 1 to 3d, the levels of CD3^+^ and CD4^+^ lymphocytes and the CD4^+^ /CD8^+^ ratio in both groups tended to decrease and then increase, and the postoperative level was close to the preoperative level in the postoperative period of 3d. The relative counts of T-lymphocyte subsets in the patients in the remimazolam group were no less than those in the propofol group.

Propofol attenuates ischemia-reperfusion injury in a partially hepatectomized liver and has a protective effect on liver function. On the one hand, the chemical structure of propofol contains a phenolic hydroxyl group, which can effectively produce anti-oxidative stress, probably related to its ability to reduce the production of reactive oxygen species (ROS), scavenge oxygen radicals, regulate the permeability of mitochondrial membranes, and inhibit lipid peroxidation [31]; on the other hand, propofol can inhibit the secretion of inflammatory cytokines through direct dilation of visceral blood vessels, alleviation of hypoxia and inadequate blood perfusion [32]. The results of this study showed that the ALT, AST, and LDH levels of patients in the remimazolam group were not higher than those in the propofol group at 1d and 3d postoperatively, and it was hypothesized that remimazolam tosylate also had a protective effect on liver function. The specific chemical structure of remimazolam tosylate causes its metabolism to be nonspecific, as it is degraded by a wide range of nonspecific tissue carboxylesterases (CES) and then excreted via the kidneys, independent of the cytochrome-dependent hepatic pathway [33]. In addition, primary human hepatocytes were cultured in a dynamic 3-D bioreactor system with continuous exposure to clinically relevant concentrations of remimazolam, and it was found that hepatocyte integrity and metabolic activity were not affected. Additionally, the expression of the CES1 gene, which is responsible for the metabolic inactivation of remimazolam, remained unaffected [34]. The effects of remimazolam tosylate on liver function at the overall and cellular levels are not fully understood and additional preclinical experiments will be required for further confirmation in the future.

Hypotension occurs from time to time during the induction period of anesthesia and is usually thought to be related to factors such as hypovolemia due to preoperative fasting as well as the vasodilatory effects of certain induction agents [35]. The patients in this study underwent laparoscopic partial hepatectomy. To reduce intraoperative bleeding, fluid intake needs to be controlled to maintain low central venous pressure. Consequently, the incidence of hypotension increases, making it crucial to carefully choose the appropriate anesthetic drugs. Blood pressure fluctuates greatly when propofol is used, and a drop in blood pressure, even if it lasts for 5 min, increases the risk of organ damage, especially in some elderly patients [36]. In contrast, the incidence of hypotension is low when remimazolam is used for the induction of anesthesia. Previous studies have shown stable arterial blood pressure and heart rate in patients sedated with remimazolam for examinations such as gastroenteroscopy and bronchoscopy [37]. Dong et al [38] showed that the incidence of hypotension during ERCP procedures was lower in patients sedated with remimazolam than in those sedated with propofol. ANTONIK et al [39] studied when 54 volunteers received a single dose of remimazolam up to 0.35 mg/kg no hypotension was reported. Yokose et al [36] studied 90 patients not less than 80 years of age scheduled for elective noncardiovascular surgery under general anesthesia and showed that the use of remimazolam contributed to hemodynamic stability during the induction of general anesthesia in elderly patients compared to the use of propofol. In the present study, we found that the suppressive effects of HR and MAP after the induction of anesthesia were also significantly milder in patients treated with remimazolam tosylate than in patients treated with propofol, suggesting that remimazolam tosylate has a greater safety profile during the induction of anesthesia, especially in elderly patients.

The following limitations of this study remain. First, the use of opioids were used in the anesthesia process, and opioids can have an effect on patient immune function and hemodynamics. Second, the patient’s age span is wide, and it is difficult to determine whether the dosages of propofol and remimazolam tosylate used for general anesthesia are equivalent or not, and the ratio of the sedative efficacy of propofol and remimazolam tosylate in general anesthesia of all ages is still not clear. Third, the present study is a single-center study, which includes only patients with Child-Pugh classification A, with a relatively small sample size, and a multicenter, large-sample-size, randomized, and double-blind trial needs to be conducted in the follow-up for the further validation of the findings of the present study.

## Conclusion

The effect of remimazolam tosylate on patients’ immune function after laparoscopic partial hepatectomy is not inferior to that of propofol, yet with a milder hemodynamic effect. Compared to propofol, Remimazolam tosylate significantly reduces the incidence of hypotension during the induction period of anesthesia, thereby exhibiting a greater safety profile.

## Data Availability

Data and materials related to this study are available from the corresponding author.

## Abbreviations

BMI: Body mass index
ASA: American Society of Anesthesiologists
ECG: Electrocardiogram
HR: Heart rate
SPO_2_: Pulse Oxygen Saturation
BIS: Bispectral index
MAP: Mean arterial pressure
CVP: Central venous pressure
VT: Tidal volume
RR: Respiratory rate
I:E: Inspiration-to-expiration ratio
P_ET_CO_2_: End-tidal carbon dioxide
PACU: Post-Anesthesia Care Unit
ALT: Alanine aminotransferase
AST: Aspartate aminotransferase
LDH: Lactic dehydrogenase
HPA: Hypothalamic-pituitary-adrenal
SNS: Sympathetic nervous system
PGE2: Prostaglandin E2
THs: Helper T-lymphocytes
CTLs: Cytotoxic T-lymphocytes
GABA: Gamma-aminobutyric acid
BDZ: Benzodiazepine
CBBS: Central benzodiazepine binding site
TSPO: Translocator protein
CRH: Corticotropin-releasing hormone
ROS: Reactive oxygen species
CES: Carboxylesterase

## References

[1] Toh MR, Wong EYT, Wong SH, Ng AWT, Loo L-H, Chow PK-H, Ngeow J (2023). Global Epidemiology and Genetics of Hepatocellular Carcinoma. Gastroenterology.

[2] Nault JC, Paradis V, Ronot M, Zucman-Rossi J (2022). Benign liver tumors. Understanding molecular physiology to adapt clinical management. Nat Rev Gastroenterol Hepatol.

[3] Peres LAB, Bredt LC, Cipriani RFF (2016). Acute renal injury after partial hepatectomy. World J Hepatol.

[4] Ypsilantis P, Simopoulos C (2016). A laparoscopic technique of partial hepatectomy in the rat. J Surg Res.

[5] Schindl MJ, Redhead DN, Fearon KCH, Garden OJ, Wigmore SJ, Edinburgh Liver S, Transplantation Experimental Research Group (2005). The value of residual liver volume as a predictor of hepatic dysfunction and infection after major liver resection. Gut.

[6] Neeman E, Ben-Eliyahu S (2013). Surgery and stress promote cancer metastasis: new outlooks on perioperative mediating mechanisms and immune involvement. Brain Behav Immun.

[7] Chang Y, Huang Y-T, Chi K-Y, Huang Y-T (2023). Remimazolam versus propofol for procedural sedation: a meta-analysis of randomized controlled trials. PeerJ.

[8] Jacobi J, Fraser GL, Coursin DB, Riker RR, Fontaine D, Wittbrodt ET, Chalfin DB, Masica MF, Bjerke HS, Coplin WM (2002). Clinical practice guidelines for the sustained use of sedatives and analgesics in the critically ill adult. Crit Care Med.

[9] Shi F, Chen Y, Li H, Zhang Y, Zhao T (2022). Efficacy and safety of Remimazolam Tosilate versus Propofol for General Anesthesia in Cirrhotic patients undergoing endoscopic Variceal Ligation. Int J Gen Med.

[10] White PF (2023). Remimazolam - can it become a cost-effective alternative to propofol for intravenous anesthesia and sedation?. J Clin Anesth.

[11] Kim R (2018). Effects of surgery and anesthetic choice on immunosuppression and cancer recurrence. J Transl Med.

[12] Yang J, Xu J, E Y, Sun T (2019). Predictive and prognostic value of circulating blood lymphocyte subsets in metastatic breast cancer. Cancer Med.

[13] Liu HZ, Deng W, Li JL, Tang YM, Zhang LT, Cui Y, Liang XQ (2016). Peripheral blood lymphocyte subset levels differ in patients with hepatocellular carcinoma. Oncotarget.

[14] Mahmoud SM, Paish EC, Powe DG, Macmillan RD, Grainge MJ, Lee AH, Ellis IO, Green AR (2011). Tumor-infiltrating CD8^+^ lymphocytes predict clinical outcome in breast cancer. J Clin Oncol.

[15] Wang K, Shen T, Siegal GP, Wei S (2017). The CD4/CD8 ratio of tumor-infiltrating lymphocytes at the tumor-host interface has prognostic value in triple-negative breast cancer. Hum Pathol.

[16] Vojdani A, Koksoy S, Vojdani E, Engelman M, Benzvi C, Lerner A (2024). Natural Killer Cells and cytotoxic T cells: complementary partners against microorganisms and Cancer. Microorganisms.

[17] Liu S, Gu X, Zhu L, Wu G, Zhou H, Song Y, Wu C (2016). Effects of propofol and sevoflurane on perioperative immune response in patients undergoing laparoscopic radical hysterectomy for cervical cancer. Med (Baltim).

[18] Wang J, Cui S, Kong L, Ma B, Gu J. Robustness of Propofol and Sevoflurane on the Perioperative Immune function of patients undergoing laparoscopic radical nephrectomy. J Oncol. 2022; 1662007. 10.1155/2022/1662007 PMC8818401 35136408

[19] Feng X, Ma Y, Yang J, Peng P, Zeng X, Shen L, Hu T, Luo Q. Comparison of effects of different anesthesia methods on immune function and liver function of liver cancer patients after operation. Biotechnol Genet Eng Rev. 2023. doi: 10.1080/02648725.2023.2201521. Online ahead of print. 10.1080/02648725.2023.2201521 37066843

[20] Yong F, Wang H, Li C, Liu W, Wang Z, Jia H (2023). Effect of sevoflurane on CD4^+^CD25^+^FOXP3^+^ regulatory T cells in patients with gastric cancer undergoing radical surgery. Cell Mol Biol (Noisy-le-grand).

[21] Liu TC (2014). Influence of propofol, isoflurane and enflurance on levels of serum interleukin-8 and interleukin-10 in cancer patients. Asian Pac J Cancer Prev.

[22] Inada T, Kubo K, Kambara T, Shingu K (2009). Propofol inhibits cyclo-oxygenase activity in human monocytic THP-1 cells. Can J Anaesth.

[23] Longhini F, Bruni A, Garofalo E, De Sarro R, Memeo R, Navalesi P, Navarra G, Ranieri G, Currò G, Ammendola M (2020). Anesthetic strategies in oncological surgery: not only a simple sleep, but also impact on Immunosuppression and Cancer Recurrence. Cancer Manag Res.

[24] Ramirez K, Niraula A, Sheridan JF (2016). GABAergic modulation with classical benzodiazepines prevent stress-induced neuro-immune dysregulation and behavioral alterations. Brain Behav Immun.

[25] Arvat E, Giordano R, Grottoli S, Ghigo E (2002). Benzodiazepines and anterior pituitary function. J Endocrinol Invest.

[26] Taupin V, Jayais P, Descamps-Latscha B, Cazalaa JB, Barrier G, Bach JF, Zavala F (1991). Benzodiazepine anesthesia in humans modulates the interleukin-1 beta, tumor necrosis factor-alpha and interleukin-6 responses of blood monocytes. J Neuroimmunol.

[27] Kim SN, Son SC, Lee SM, Kim CS, Yoo DG, Lee SK, Hur GM, Park JB, Jeon BH (2006). Midazolam inhibits proinflammatory mediators in the lipopolysaccharide -activated macrophage. Anesthesiology.

[28] Joo HK, Oh SC, Cho EJ, Park KS, Lee JY, Lee EJ, Lee SK, Kim HS, Park JB, Jeon BH (2009). Midazolam inhibits tumor necrosis factor-alpha-induced endothelial activation: involvement of the peripheral benzodiazepine receptor. Anesthesiology.

[29] Ruff MR, Pert CB, Weber RJ, Wahl LM, Wahl SM, Paul SM (1985). Benzodiazepine receptor-mediated chemotaxis of human monocytes. Science.

[30] Jin Z, Mendu SK, Birnir B (2013). GABA is an effective immunomodulatory molecule. Amino Acids.

[31] Hao W, Zhao Z-H, Meng Q-T, Tie M-E, Lei S-Q, Xia Z-Y (2017). Propofol protects against hepatic ischemia/reperfusion injury via miR-133a-5p regulating the expression of MAPK6. Cell Biol Int.

[32] Kumakura S, Yamaguchi K, Sugasawa Y, Murakami T, Kikuchi T, Inada E, Nagaoka I (2013). Effects of nitrous oxide on the production of cytokines and chemokines by the airway epithelium during anesthesia with sevoflurane and propofol. Mol Med Rep.

[33] Wang M, Zhao X, Yin P, Bao X, Tang H, Kang X (2022). Profile of Remimazolam in Anesthesiology: a narrative review of Clinical Research Progress. Drug Des Devel Ther.

[34] Freyer N, Knöspel F, Damm G, Greuel S, Schneider C, Seehofer D, Stöhr T, Petersen K-U, Zeilinger K (2019). Metabolism of remimazolam in primary human hepatocytes during continuous long-term infusion in a 3-D bioreactor system. Drug Des Devel Ther.

[35] De Wit F, Van Vliet A, De Wilde R, Jansen J, Vuyk J, Aarts L, De Jonge E, Veelo D, Geerts B (2016). The effect of propofol on hemodynamics: cardiac output, venous return, mean systemic filling pressure, and vascular resistances. Br J Anaesth.

[36] Yokose M, Takaki R, Mihara T, Saigusa Y, Yamamoto N, Masui K, Goto T (2022). Hypotension after general anesthesia induction using remimazolam in geriatric patients: protocol for a double-blind randomized controlled trial. PLoS ONE.

[37] Sneyd JR, Gambus PL, Rigby-Jones AE (2022). Current status of perioperative hypnotics, role of benzodiazepines, and the case for remimazolam: a narrative review. Br J Anaesth.

[38] Dong S-A, Guo Y, Liu S-S, Wu L-L, Wu L-N, Song K, Wang J-H, Chen H-R, Li W-Z, Li H-X (2023). A randomized, controlled clinical trial comparing remimazolam to propofol when combined with alfentanil for sedation during ERCP procedures. J Clin Anesth.

[39] Antonik LJ, Goldwater DR, Kilpatrick GJ, Tilbrook GS, Borkett KM (2012). A placebo- and midazolam-controlled phase I single ascending-dose study evaluating the safety, pharmacokinetics, and pharmacodynamics of remimazolam (CNS 7056): part I. Safety, efficacy, and basic pharmacokinetics. Anesth Analg.

